# Analysis of trabecular distribution of the proximal femur in patients with fragility fractures

**DOI:** 10.1186/1471-2474-14-130

**Published:** 2013-04-09

**Authors:** Yaogang Lu, Lei Wang, Yongqiang Hao, Ziping Wang, Minghui Wang, Shengfang Ge

**Affiliations:** 1 Department of Orthopedics, Pudong New Area District Zhoupu Hospital, Shanghai 201318, People’s Republic of China; 2 Department of Orthopaedics, Ninth People’s Hospital, Shanghai JiaoTong University School of Medicine, 639 Zhizaoju Road, Shanghai 200011, People’s Republic of China; 3 Department of Ophthalmology, Ninth People’s Hospital, Shanghai Jiaotong University School of Medicine, 639 Zhizaoju Road, Shanghai 200011, People’s Republic of China

**Keywords:** Osteoporosis, Hip fracture, Computed tomography (CT), Proximal femur, Trabecular pattern

## Abstract

**Background:**

Multi-detector computed tomography (MDCT) was used in order to assess the trabecular distribution of proximal femur and its relationship with hip fragility fractures.

**Methods:**

A total of 99 elderly women were scanned by MDCT including: 27 trochanteric hip fractures (group A), 40 femoral neck fractures (group B), and 32 non-fractures (group C). A mid-coronal MPR image of the proximal femur was reconstructed for every patient by e-Film95 software. Four regions of interest (ROI) were chosen in the images including compressive trabecula (ComT), tensile trabecula (TenT), trochanteric trabecula (TroT) and Ward's triangle (WT) region. The mean CT values were measured by the software.

**Results:**

The mean age was 81.44, 74.10 and 69.25 years for groups A, B and C, respectively. There was significant inter-group differences based on one-way ANOVA (P<0.05). The CT values in the four ROIs had significant differences in the groups except for TenT between group A and B (P>0.05). After the age adjustment with ANCOVA, the mean CT values of TroT and WT were significantly lower in group A as compared to that of the group B (P<0.05). However, there were no significant differences for ComT and TenT between groups A and B (P>0.05).

**Conclusions:**

The occurrence of femoral neck fracture was closely related to the degeneration of ComT and TenT. Trochanteric hip fractures were associated with a more severe degeneration in TroT as well as an enlargement of WT region besides the ComT and TenT degeneration. We concluded that the hip fragility fractures might be predicted by the measurement of the mean CT values in ComT, TenT, TroT and WT region.

## Background

The elderly population is increasing as the lifespan is extended. The elderly may develop fragility fractures after minor injuries, the most common of which is a hip fracture. Hip fragility fractures are defined [1] as the fracture that are caused by sideway falls from a standing height. They have a slow initial velocity on the greater trochanter and mainly include trochanteric hip and femoral neck fractures.

The impact forces on the hip of the elderly people during the falls from a standing height, on average, exceed the strength of their femurs by approximately 50% [2]. A low bone mineral density (BMD) is the ultimate cause for hip fragility fractures. Dual-energy x-ray absorptiometry (DXA) is considered the gold standard technique for assessing the bone health. Hip fragility fractures are closely related to the lower BMD values [3]; however, the BMD values vary across fractures and non-fractures. Currently, the women at high risk for hip fragility fractures cannot be identified by measurement of the BMD with DXA.

The trabecula and cortex are two critical components, which determine hip fracture resistance. However, the current technology is limited in its ability to measure cortical thickness, especially in the sub-millimeter range, which lies within the point spread function of the contemporary clinical scanners [4]. Therefore, the fracture risk cannot be evaluated by measurement of the cortical thickness. It has been shown that cancellous greatly contributed to the proximal femur bone strength [5]. Although DXA is commonly used for measurement of the BMD, it cannot distinguish between the cortical and trabecular bone compartments. Quantitative computed tomography (QCT) creates three-dimensional images that enable volumetric evaluation of bone mineral content and permits separate assessment of trabecular and cortical bone density. Furthermore, it has been shown that QCT provided a true assessment of the BMD [6]; however, the trabecular distribution in the proximal femur was extremely asymmetric and a different distribution resulted in a different mechanism [7]. When evaluating fracture risk, trabecular distribution must be taken into account. However, commercially available QCT only measures the quantity of trabecula and cannot distinguish their distribution, which limits its application in hip fracture risk evaluation. There is no effective method for analyzing trabecular distribution in the proximal femur; therefore, the questions regarding the distribution of trabecula in the proximal femur have not been adequately answered.

Although the distribution of trabecular bone in the proximal femur is extremely asymmetric, it has some specific characteristics. There are five trabecular groups in the proximal femur including: principal compressive; principal tensile; secondary compressive; secondary tensile and greater trochanteric trabeculae. The elastic modulus of the trabecular bone material has been investigated in many studies at the tissue level. The elastic modulus of the trabecular bone tissue has been suggested to be close to that of the cortical bone tissue [8]. It is well known that osteoporosis could be diagnosed by trabecular pattern changes observed in the proximal femur radiographs based on the Singh Index grading system. Since five trabecular groups are located in the internal side of the proximal femur, we were unable to accurately detect their characteristics from one-dimensional radiographs. Therefore, it was considered too variable for the diagnosis [9].

In recent years, multi-detector computed tomography (MDCT) has been widely applied in many orthopedic fields. Since CT allowed a non-invasive evaluation of internal morphology and had a comparable value with histologic thin sections of cancellous bone, some researchers [10] have successfully characterized the trabecular structure parameters by MDCT.

In this study, patients with hip fragility fractures were selected for analysis. A mid-coronal image of the proximal femur on the unaffected side was reformatted from the MDCT data. The mean CT values were measured from these images in three trabecular and Ward’s triangle regions. We used this technique to characterize the trabecular pattern changes in the proximal femur in order to evaluate the clinical risk factors, which were associated with hip fragility fractures, and to offer a potential method for hip fragility fracture prediction and prevention.

## Methods

### Subjects

We studied 99 elderly women including: 27 trochanteric hip fractures (group A; mean age of 81±7 years); 40 femoral neck fractures (group B; mean age of 74±10 years) and 32 non-fractures (group C; mean age of 69±9 years). All of the patients were older than 55 years of age and were reffered to our hospital due to a sideways fall from a standing height on the greater trochanter. The exclusion criteria included a history of generalized bone disease, malignant disease and any drug treatment, which could have impacted the bone metabolism. Our study was approved by the new Pudong Area District Zhoupu Hospital Ethics Committee. All the patients who enrolled in the study provided informed consent and the study was conducted according to the guidelines approved by the ethical permission.

### CT scan of the patient

The CT scans were performed on a Toshiba Active 16 detector system (Toshiba Medical Systems Corporation, Japan). Both of the hips were scanned (helical mode: pitch, 1.375:1; 135 kV; 250 mAs; 10 mm beam width; 16 channels; matrix 512×512) from the superior aspect of the femoral head up to 3 cm distal to the trochanter with the two lower limbs fixed in the neutral position. Hip transverse sections were reconstructed at an interval of 1 mm.

### Image reformation

**Figure 1:**
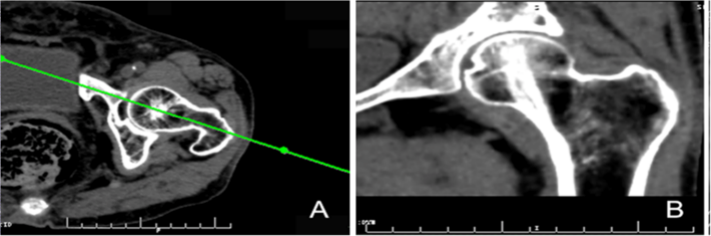
**Reformating the mid-coronol image of proximal femur.** (**A**) The Transverse section of femoral neck. The green line is the center of the femoral neck; (**B**) A mid-coronol MPR image of proximal femur is reformatted through the green line.

**Figure 2:**
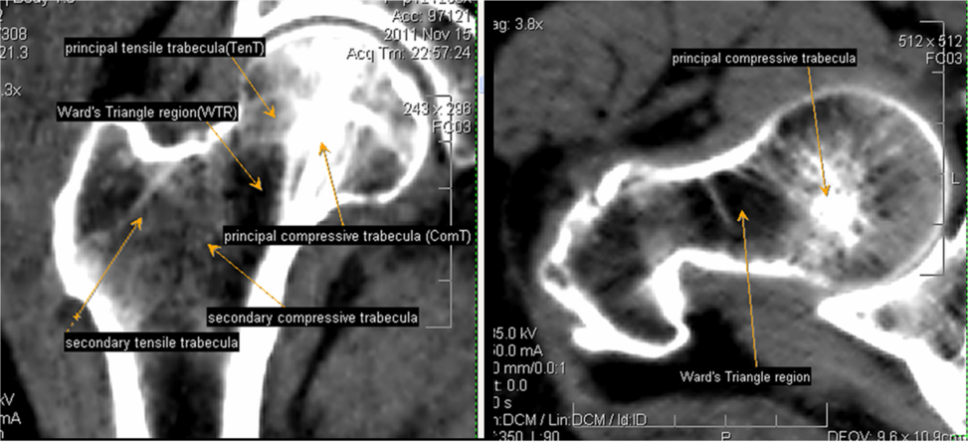
**The location of the trabeculae and Ward's Triangle region internal proximal femur.** (**A**) A mid-coronol image of proximal femur reveals: principal compressive trabecula; principal tensile trabecula; secondary compressive trabecula; secondary tensile trabecula and Ward’s Triangle region. (**B**) A transverse section of femoral neck reveals: principal compressive trabecula and Ward's Triangle region.

The image processing was done by e-Film95 software (Merge Healthcare, Chicago, IL, USA). We imported the CT data into the software and set the window width and level at 350 and 90 HU, respectively. The unaffected hip was selected to measure four parameters. A center line of the femoral neck was drawn in transverse section of the femoral neck (Figure 1A). A two-dimensional mid-coronal image of the proximal femur (Figure 1B) was reconstructed along the center line by multi-planar reformation (MPR) technology. This reformatted image was a mid-coronal image of the proximal femur and showed the trabecular bone structure of the internal proximal femur. The principal compressive trabecula, principal tensile trabecula, secondary compressive trabecula, secondary tensile trabecula and Ward's triangle region could be clearly observed on this image (Figure 2).

### Region of interest (ROI) selection

Four regions of interest (ROI) were manually selected in the reformatted mid-coronal image (Figrue 3). Two ellipses (major axis: 2.3 cm; area: 1.4 cm^2^) were chosen in \the region of principal compressive trabecula (ComT) and Ward's triangle (WT). A circular ROI (area: 0.5 cm^2^) was chosen in principal tensile trabecula (TenT). In order to select the trochanteric trabecula (TroT) ROI, we drew two lines along the center and the based on the femoral neck, and one circular ROI (area: 3.1 cm^2^) was chosen outside the intersection of these two lines. The TroT ROI contained the secondary compressive trabecula and secondary tensile trabecula. After four ROIs were chosen, e-Film95 software automatically displayed their mean CT values, which was measured in Hounsfield units [HU] in the image. All the processes were performed by one author at three different times and the averaged values were regarded as the final results.

### Statistical analysis

Statistical analyses were performed using SPSS (version 13.0, Chicago, IL, USA). All tests were done using a two-sided 0.05 level of significance. The mean and standard deviation (SD) for each of the listed parameters were calculated and the data was applied to tests of normality with Shapiro-Wilk method. Inter-group comparisons were performed for age and the mean CT values of the four ROIs using one-way ANOVA. Nonparametric tests were performed to compare the difference in the groups, where the data was not normally distributed. Analysis of covariance (ANCOVA) was performed for four parameters with age adjustment.

## Results

**Figure 3:**
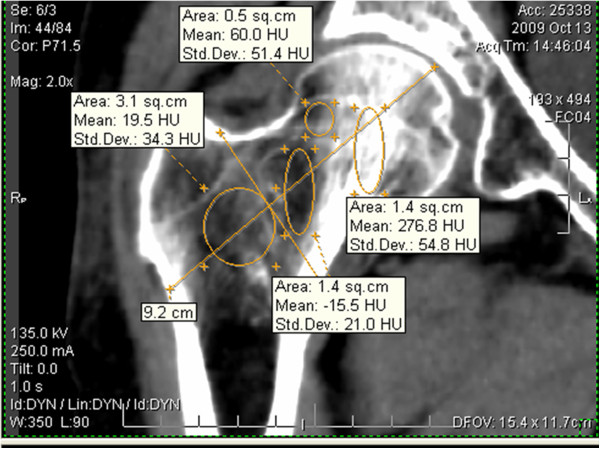
**Four regions of interest (ROI) and the axes of the coordinate system used to position them. A**=Compressive trabecula ROI (ComT); **B**=Tensile trabecula ROI (TenT); **C**=Trochanteric trabecula ROI (TroT); **D**= Ward's triangle ROI (WT); **E**=The center of femoral neck and F=The base of femoral neck.

We found that the distribution of trabeculae in the proximal femur was extremely asymmetric. ComT was located at the center of the femoral head in the transverse sectional image of the the femoral head (Figure 2B). In the reformatted mid-coronal image of the proximal femur (Figure 2A), the ComT extended from the femoral neck inferior cortex to the superior aspect of the femoral head. Likewise,the TenT extended from the femoral neck superior cortex to the inside of the femoral head. They further intercrossed in the center of the femoral head (Figure 2A). ComT was wider than TenT and had a higher density. In a healthy individual, the mean CT value of ComT exceeded 400 HU, which was close to the density of the cortex. The mean CT value of TenT was <200 HU, which was close to the density of the cancellous. The mean CT values of ComT and TenT were higher than that of the surrounding trabeculae in the proximal femur. The WT region was located outside of ComT and was more like an ellipse (Figures 2, 3). Secondary compressive trabeculae and tensile trabeculae were also shown in the reformatted image (Figure 2A), but the greater trochanteric trabeculae could not be clearly detected.

**Figure 4:**
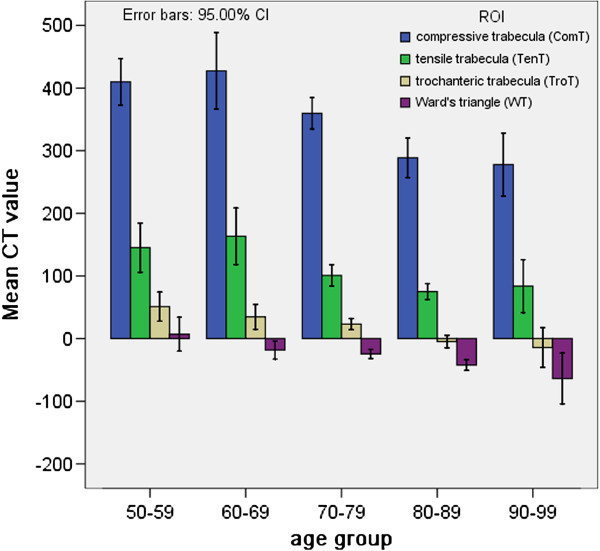
**The mean CT values of four ROIs in five different age (50–99 years) groups.** We found that CT values of four ROIs significantly decreased for the patient over 70 years old.

**Figure 5:**
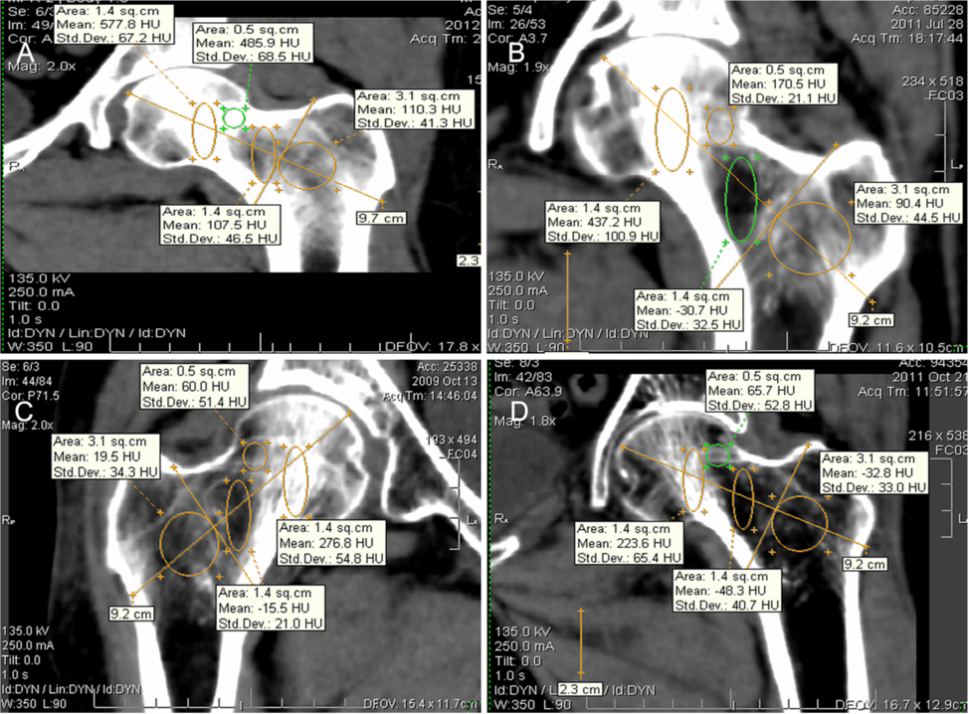
**The mean CT values of four ROI for four patients with different age.** (**A**) A 45-year-old middle age woman; (**B**) A 67-year-old no-fracture patient; (**C**) A 75-year-old woman with femoral neck fracture and (**D**) A 82-year-old woman with trochanteric hip fracture.

The trabecular bone structure of the proximal femur was degenerated with age and the mean CT value gradually decreased in the four ROIs with age. As shown in Figure 4, the mean CT values were different for the four ROIs with different age groups. We found that CT values were significantly decreased for the patients over 70. In Figure 5, the mean CT values of the four ROIs are shown in four people with different ages. Figure 5A was related to a 45-year-old middle age woman, where the mean CT values for ComT, TenT, TroT and WT were 577.8, 485.9, 110.3 and 107.5 HU, respectively. Figure 5B was related to a 67-year-old no-fracture woman who suffered from a sideways fall and the mean CT values of her four ROIs were 437.2, 170.5, 90.4 and 30 HU. Furthermore, there was a mild trabecular degeneration in her proximal femur. Figure 5C was related to a 75-year-old woman with a femoral neck fracture and the mean CT values of her four ROIs were 276.8, 60, 19.5 and −15.5 HU. Compared with no-fracture group, the mean CT values of the three trabecular ROIs were significantly lower. Figure 5D was related to an 82-year-old woman with trochanteric hip fracture. Compared to the femoral neck fracture group, the mean CT values of her TroT (−32.8 HU) and WT (−48.3 HU) were significantly lower.

A significant inter-group difference existed when the ages were compared (Table 1) among the three groups (P<0.05). The mean, standard deviation, standard error and minimum and maximum CT values for the three groups are shown in Table 2 for the four ROIs. The mean CT values of ComT, TenT, TroT and WT were 285.11, 73.26, -19.48 and −52.22 HU in group A; and 325.15, 88.25, 23.55 and −23.03 HU for group B; and 435.16, 154.22, 44.06 and −8.09 HU for group C. The mean CT values for four ROIs were applied to the test of normality and the results indicated that the data was normally distributed except for the TroT and WT ROIs in group C. The LSD test and one-way ANOVA were used in order to compare the inter-group differences. Except for TroT and WT ROIs of group C (Table 3), we found that there was significant inter-group differences with the exception of TenT between groups A and B (p=0.222). Non-parametric statistics for TroT and WT ROIs were performed and demonstrated that there was significant differences in the groups.

**Table 1:** Mean±SD, standard deviation and standard error of age in three groups and p values for inter group discriminations

**Group**	**Number**	**Mean**	**Std. deviation**	**Std. error**	**p Values**		
					**vs. A**	**vs. B**	**vs. C**
A	27	81.44	6.773	1.303		0.002	0.000
B	40	74.10	10.160	1.606	0.002		0.026
C	32	69.25	9.284	1.641	0.000	0.026	
Total	99	74.54	10.142	1.019			

Group A: trochanteric hip fractures; Group B: femoral neck fractures; Group C: non-fractures. P values for LSD t-tests inter-group using one-way ANOVA.

**Table 2:** Mean, standard deviation, standard error, minimum and maximum CT values for four regions of interest in the three groups

**ROI**	**Group**	**Number**	**Mean (HU)**	**Std. Deviation (HU)**	**Std. Error (HU)**	**Minimum (HU)**	**Maximum (HU)**
**Compressive trabeculae**	A	27	285.11	69.389	13.354	158	400
	B	40	325.15	77.113	12.193	170	566
	C	32	435.16	59.994	10.605	316	531
	Total	99	350.80	93.549	9.300	158	566
**Tensile trabeculae**	A	27	73.26	31.478	6.058	15	135
	B	40	88.25	36.794	5.818	25	183
	C	32	154.22	69.978	12.370	53	316
	Total	99	106.29	59.692	5.974	15	316
**Trochanteric trabeculae**	A	27	−19.48	14.151	2.723	−42	6
	B	40	23.55	27.200	4.301	−37	98
	C	32	44.06	30.115	5.324	6	115
	Total	99	18.44	35.435	3.561	−42	115
**Ward’s Triangle**	A	27	−53.22	21.497	4.137	−100	−17
	B	40	−23.03	22.955	3.630	−70	16
	C	32	−8.09	35.443	6.266	−55	97
	Total	99	−26.43	32.279	3.244	−100	97

Group A: Trochanteric hip fractures; Group B: Femoral neck fractures; Group C: Nonfractures.

**Table 3:** Values of statistical significance for CT values in four regions of interest in the three groups before and after adjustment for age

**Group**		**Compressive trabeculae**		**Tensile trabeculae**		**Trochanteric trabeculae**		**Ward's Triangle**	
		**p**	p*	**p**	p*	**p**	p*	**p**	p*
**A**	**B**	.024	.071	.222	.667	.000	.000	.000	.000
	**C**	.000	.000	.000	.000				
**B**	**A**	.024	.071	.222	.667	.000	.000	.000	.000
	**C**	.000	.000	.000	.000				
**C**	**A**	.000	.000	.000	.000				
	**B**	.000	.000	.000	.000				

Group A: Trochanteric hip fractures; Group B: Femoral neck fractures; Group C: Non-fractures. P values using LSD test and one-way ANOVA. p* values after adjustment for age using ANCOVA.

ANCOVA was performed by Bonferroni test with age adjustment in order to exclude the influence that was imposed on the three groups by age differences except for TroT and WT ROIs of group C (Table 3). The CT values of ComT and TenT had no significant differences between groups A and B (p=0.71 and p=0.667, respectively). However, there was significant difference in TroT and WT between this two groups (p=0.000 and p=0.000, respectively). There were also significant differences between groups C and A or C and B in terms of ComT and TenT.

## Discussion

The aging population is significantly increasing and the exponential increase in hip fractures with aging imposes a substantial and increasing health burden. Although DXA and QCT are commonly used to evaluate the risk of hip fragility fractures, they are unable to correctly forecast the occurrence of hip fragility fractures. Hip fragility fractures take place mostly in the region of the inter-trochanter and subcapital of the femoral neck, where there is a shortage of cortex. Therefore investigation of the trabeculae internal proximal femur has higher scientific value for fracture risk evaluation. In this study, we applied MPR technology to reconstruct a mid-coronal image of the proximal femur with a high quality image that was acquired by MDCT and measured the mean CT values of four ROIs in the internal proximal femur. The reformatted mid-coronal image of the proximal femur was similar to a mid-coronal section through the center of proximal femur, and clearly showed the distribution of the trabeculae internal proximal femur and WT region. This method was applied for the first time and was able to analyze the distribution of trabeculae internal proximal femur as compared to the DXA and QCT.

Based on the evidence provided from a high-speed video of simulated fractures, Bakke et al. [11] demonstrated that during the sideways fall, the proximal femur fractures are initiated in the superolateral cortex. The tensile trabeculae extends from the superior cortex of the femoral neck and plays an important role in the occurrence of femoral neck fractures. Among normal individuals, TenT was thinner than ComT with mean CT values of approximately one-half of that of the ComT (Figure 3), which withstood the tensile stresses in physiologic loading conditions. During a sideways fall on the greater trochanter, the TenT often withstands compressive stresses. When the TenT becomes degenerates and the impact forces exceed the elastic modulus, the femoral neck fractures occur. In this study, we found that ComT and TenT were significantly degenerated in group B and the mean CT values were significantly lower than that of the group C (Tables 2 and 3). This result indicated that ComT and TenT mean CT value evaluation might help to discriminate the femoral neck fractures from non-fractures.

The hip fragility fractures include femoral neck and trochanteric hip fractures. All the fractures result from sideway falls on the greater trochanter; however, the reason why the same mechanism leads to different fractures is not well understood. In this study, we demonstrated that the CT values of ComT, TenT and TroT were significantly lower in groups A and B as compared to those of the group C with or without adjustment for age. Therefore, it was concluded that the trabecular degeneration played an important role in all kinds of hip fragility fractures. The CT values of ComT and TenT (285.11 and 72.26 HU, respectively) were lower in group A as compared to that of the group B (325.15 and 88.25 HU, respectively), but there was no significant difference between groups A and B after age adjustment (Table 3). When the CT values of TroT and WT region were compared between groups A and B with or without adjustment for age (Table 3), the mean CT values of TroT and WT was significantly lower for group A (−19.48 and −53.22 HU, respectively) as compared to that of the group B (23.55 and −23.03 HU, respectively). This study indicated that the trochanteric fractures had more severe degeneration at the inter-trochanter than that of the femoral neck fractures (Figure 4C and D). Therefore, during a sideways fall on the greater trochanter, the more degenerated TroT would be broken first, which can result in trochanteric hip fractures. This might be the reason why trochanteric hip fractures occur more often in elderly patients.

The CT values for water and trabeculae were about 0 HU and 200 HU, respectively. Therefore, if the mean CT value of one ROI was less than 0 HU, its density was close to the density of soft tissue. In this study, the CT values for the TroT and WT region were −19.48 and −53.22 HU in group A, respectively. Therefore, the patients with trochanteric fractures must have had severe trabecular degeneration that was resulted in TroT region to be filled with yellow bone marrow. On this basis, the analysis of TroT and WT regions could help physicians to evaluate the risk of trochanteric hip fractures.

Because the states of CT detectors may vary with temperature, humidity and circumstances, this could lead to CT value floating. When CT images are used to measure the BMD, a phantom is recommended for calibration purposes. However, recent studies [12] have reported results from a phantomless QCT BMD system with robust clinical utility for the detection of reduced BMD. Recently, several clinicians [13,14] have directly measured the mean CT values of the tissues to determine their density. In fact, because the CT values were regularly calibrated, their floating degree of was limited to ±5 HU, which posed no effect on the accuracy of the measurement. The results of our study further confirmed that the measurement of CT values was useful and could be considered as a BMD indictor. Moreover, the method was easy to be implemented in practice. However, the following points should be considered to assure the accuracy of the measurement: 1) The two lower limbs must be fixed in a neutral position with the hip and knee joints unbent when patients are scanned; 2) The original CT data should be reconstructed with an interval of 1 mm in order to improve the resolution of MPR images; 3) The hardware might interfere with the MDCT scan and influence the quality of the image, therefore the patient should be scanned pre-operatively; and 4) When the mid-coronal image of the proximal femur is reconstructed, we must assure that the image is the center section of the proximal femur. Our method was based on MDCT scan and was associated with higher radiation doses. Several studies [15] have shown that MDCT delivered doses of 1–3 mSv to the patients for evaluation of hip structure. Therefore, low- dose protocols were needed to reduce the radiation exposure and minimize the health risks. In order to clearly visualize individual trabeculae, one should set the window width and level to 350 and 90 HU, which were close to the CT value range of trabeculae. Furthermore, the matrix image resolution should be set to 512×512 with a 1-mm slice thickness. Marrow fat and subchondral sclerosis might lead to some errors in the measurement. Therefore, the patients with a history of generalized bone disease, malignant disease and any drug treatment should be excluded.

## Conclusion

In summary, we developed a new method for analyzing the distribution of trabeculae internal proximal femur by MDCT. The results showed that MDCT might have the potential to characterize the trabecular pattern and distribution of internal proximal femur as a simple and precise methodology. Trabecular degeneration plays an important role in the occurrence of hip fragility fractures. Femoral neck fractures have a close relationship with the degeneration of ComT and TenT. Patients with trochanteric hip fractures have more severe degenerations in their TroT and an enlargement of WT region in addition to the degeneration of ComT and TenT. Therefore, the risk of hip fragility fractures could be evaluated by measurement of mean CT values in ComT, TenT, TroT and WT regions.

## Competing interests

The authors declared that they had no competing interests.

## Authors’ contributions

YL and LW were the main authors, contributing equally to this work. ZW and MW conducted the analysis and interpreted the data. YH and SG revised the manuscript for intellectual content. All authors read and approved the final manuscript.

## Pre-publication history

The pre-publication history for this paper can be accessed here:

http://www.biomedcentral.com/1471-2474/14/130/prepub
